# Supplementary material for: MaAsLin 3: refining and extending generalized multivariable linear models for meta-omic association discovery
Source: Nat Methods. 2026 Jan 15;23(3):554–64. doi: 10.1038/s41592-025-02923-9 (PMC12982127; doi:10.1038/s41592-025-02923-9)
Supplement: Supplementary file 1 — Supplementary Figs. 1–4, Supplementary Information and Supplementary Methods. [file 41592_2025_2923_MOESM1_ESM.pdf]

nature methods

Supplementary information

https://doi.org/10.1038/s41592-025-02923-9

MaAsLin 3: refining and extending generalized multivariable linear models for meta-omic association discovery

In the format provided by the authors and unedited

Supplementary Information

Prevalence modeling in prior tools

Prior methods account for zero inflation with different strategies. In ALDEx2, a pseudo-count of 1/2 is used for each taxon in each sample in the Bayesian prior. In ANCOM-BC2, zeros are split into outlier zeros (treated as missing data), structural zeros (taxa that are always zero in one group, marked as differentially abundant), and sampling zeros (replaced with pseudocounts). When pseudo-counts are used, ANCOM-BC2 also tests the model's sensitivity to the magnitude of the pseudo-count. MaAsLin 2 also uses a pseudo-count for zeros, setting them to half the minimum observed relative abundance per feature. Importantly, apart from ANCOM-BC2's handling of structural zeros, these methods only test whether feature prevalence (presence or absence) is associated with covariates insofar as the prevalence association contributes to the abundance association. Some more niche methods explicitly separate prevalence and abundance testing(1; 2), but they either do not handle multiple covariates or do not give effect sizes in addition to significance tests.

Effect size accuracy

When comparing the modeled coefficients from each method to the true abundance coefficients, all methods produced coefficients biased towards zero, but MaAsLin 3 and ANCOM-BC2 yielded the least shrinkage (**Fig. 1C**). When methods impute zeros with simple pseudo-counts, the fit coefficients become a combination of the true effect and a null effect (zero slope) due to high random sparsity in the data. Despite the shrinkage, with at least 50 samples, all methods produced coefficients that showed moderate to high Spearman correlations with the true abundance coefficients (averages 0.44-0.88), though MaAsLin 3 and ANCOM-BC2 performed slightly better than the rest at high sample sizes (average 0.85 and 0.83 versus 0.60 and 0.47 for MaAsLin 2 and ALDEx2 at 1000 samples) (**Fig. 1C**). Thus, though the coefficients are biased towards zero in models that rely heavily on pseudo-counts, the ordering of the effect sizes is generally accurate.

When varying the effect size of the true coefficients, MaAsLin 3 and ALDEx2 retained the highest precision across all effect sizes (average precision $\geq$ 0.85 and $\geq$ 0.96), with MaAsLin 2 losing precision at high effect sizes and ANCOM-BC2 having low precision at all effect sizes (average precision $\leq$ 0.55; **Extended Data Fig. 3**). ANCOM-BC2 had slightly higher recall at very low effect sizes (average 0.08 with effect sizes 0.5-1), but MaAsLin 2 and MaAsLin 3 had the highest recall with effect sizes above 2.5. Coefficients fit by the models were shrunk more when the true coefficients were larger, and, for each method, the correlation between the fit and true coefficients was similar for any true effects larger than 1.

Remaining inaccuracies are attributable to finite read depth

Despite the improvements of MaAsLin 3 over previous methods and simpler regressions, precision and recall remained imperfect at high sample sizes, even under otherwise ideal simulations (**Extended Data Fig. 10A**). To investigate the remaining error, we increased the read depth from 50,000 to 50,000,000 reads (in such simulations, each read is equally informative, akin to a marker gene sequencing read). While this ultra-deep read depth is impractical in e.g. most 16S experiments, it serves to demonstrate that the remaining

inaccuracies in MaAsLin 3, at least in well-characterized communities, are due to the limitations of finite read depth. In this ultra-deep sequencing scenario, the precision of the MaAsLin 3 prevalence modeling and the abundance modeling with spike-in normalization increased to the nominal 0.9 level (average precision 0.93 and 0.92; **Extended Data Fig. 10B**). The recall of the prevalence and abundance models (both with and without spike-in normalization) increased to nearly 1 (all models above 0.85 on average); the relative shrinkage error was almost eliminated (all shrinkages at worst -8.4% on average); and the Spearman correlations increased to nearly 1 (all at least 0.83 on average). These improvements reflect the dual benefits of (1) reducing variability in the observed feature abundance due to variability in which reads happen to be sequenced and (2) reducing the probability that a feature truly present will not be detected due to none of its reads being sequenced. However, these improvements were not as consistent or substantial for other DA methods (**Extended Data Fig. 10C**).

MaAsLin 3's linear model extensions enable new experimental designs

In addition to its substantial model extensions to differentiate prevalence and abundance associations, MaAsLin 3 enables five other new types of inference (**Table 1**). First, expanding on the random intercept component of MaAsLin 2, MaAsLin 3 allows the specification of general mixed effects models, including those with interaction terms among metadata covariates. Second, MaAsLin 3 allows testing for omnibus differences among three or more levels of a covariate, expanding categorical testing protocols beyond binary comparisons (**Methods**). When evaluated on SparseDOSSA 2 synthetic data (**Methods**), the omnibus test shows moderate precision (average precision $\geq$ 0.69) across sample sizes ranging from 20 to 500, with higher precision in the most common 50-100 single study sample range (**Sup. Fig. 1A**). Third, MaAsLin 3 enables testing for level-versus-level differences in ordered predictors, such as dietary frequency data or disease progression (**Methods**). Again when evaluated using SparseDOSSA 2, with sample sizes of 50 or larger, this ordered predictor option maintained high precision (average $\geq$ 0.86; **Sup. Fig. 1B**). The difference in the precision can be attributed to the fact that the omnibus test does not explicitly account for compositionality, while the ordered predictor option does, using the median comparison strategy as above. Fourth, MaAsLin 3 can perform contrast tests among the fit coefficients, which can be useful when testing all pairwise differences among categorical variables (**Methods**). Fifth, MaAsLin 3 natively allows the specification of a feature-specific covariate, such as gene DNA abundance when regressing gene RNA abundance, an important control in metatranscriptomics experiments(3).

In particular, MaAsLin 3's feature-specific covariate testing directly implements our recommended best practices for differential expression testing in any metatranscriptomics analysis(3). To demonstrate this, we applied these improvements to bioBakery 3 profiles from the HMP2 IBDMDB metatranscriptomes(4; 5). Paired metatranscriptomic and metagenomic samples (383 samples from 52 individuals with Crohn's disease (CD), 234 samples from 30 individuals with ulcerative colitis (UC), and 200 samples from 27 individuals without IBD) were used to analyze expression of microbial pathways—coordinated biochemical reactions for a biological function (**Sup. Fig. 2**). In particular, pathway RNA relative abundances were regressed on the corresponding pathways' DNA relative abundances (functional potential) in addition to disease status and dysbiosis, while controlling for age, antibiotic usage, read depth, and repeated sampling (**Methods**). Of the 209 significant diagnosis or dysbiosis associations identified (no model fitting errors, q-

value < 0.1), 142 (68%) were abundance associations, suggesting that while pathway expression varies in both abundance and prevalence, it predominantly differs in abundance (once controlled for functional potential).

To additionally demonstrate MaAsLin 3's group and ordered covariate testing, all IBD-MDB metagenomes from participants with CD ($n = 750$) were used to regress species abundance and prevalence on food consumption frequency data. These were applied to both MetaPhlAn 4(6) (recent) and MetaPhlAn 3(5) profiles (as used in the IBDMDB publication; see **Methods**). Consistent with the group and ordered covariate models testing similar but distinct hypotheses in this setting, 59 of the 132 significant ordered associations had a corresponding group-wise association, and 59 of the 107 significant group-wise associations had a corresponding significant ordered association (MetaPhlAn 4). Of the 797 diet associations discovered in either MetaPhlAn 3 and MetaPhlAn 4, only 28 overlapped between the two, a result of differences in the abundance assigned to known taxa, differences in which taxa were included in each classifier, and differences in updated taxonomic naming. However, among the associations that did overlap, there was high agreement in the magnitude of the association (**Sup. Fig. 3**). One such association was the positive association between starch consumption in the last 4-7 days and *Eubacterium ventriosum* (SGB5045) prevalence, which is consistent with previous reports of reductions in other *Eubacterium* species during high protein, low carbohydrate diets that are depleted of starch(7; 8). Thus, MaAsLin 3's linear model extensions expand the scope of inference in microbiome studies and identify plausible associations in diet data.

HMP2 results

Extending a previous analysis(4) to account for differences in both the pediatric gut microbiome and IBD phenotypes, we stratified the IBDMDB cohort into populations that were pediatric (age < 16; 284 CD samples from 23 individuals, 95 UC from 8 individuals, and 161 non-IBD from 11 individuals) or adult (age ≥ 16; 448 CD samples from 37 individuals, 363 UC from 29 individuals, and 267 non-IBD from 16 individuals), with 16 corresponding to an age by which all participants had likely entered puberty(9; 10). The identified microbial biomarkers tended to show variable positive associations with IBD dysbiosis and diagnosis by age but more consistent negative associations. Of the 60 positive associations from either population (false discovery rate (FDR) q-value < 0.1, $|\beta| > 1$, and no model-fitting errors), only two overlapped (see **Methods** for criteria) between populations: *Citrobacter freundii* (SGB10083) and *Klebsiella pneumoniae* (SGB10115) with CD dysbiosis, both of which are thought to drive gut inflammation(11; 12; 13).

Among the non-overlapping associations, 35 were unique to the adult population, with several microbes enriched in CD including several species of *Enterocloster* (*E. bolteae* (SGB4758), *E. aldenensis* (SGB4762), *E. lavalensis* (SGB4725), *E. clostridioformis* (SGB4760), and *E. citroniae* (SGB4761)) and several species from the class *Clostridia* (e.g. *Flavonifractor plautii* (SGB15132) and *Blautia faecicola* (SGB4867); **Fig. 4A**). Several well known IBD-associated microbes were positively associated with adult CD dysbiosis including *Enterococcus faecium* (SGB7967) and *E. faecalis* (SGB7962)(14; 15; 16), *Klebsiella oxytoca* (SGB10118)(17), and *Hungatella hathewayi* (SGB4741), a microbe with substantial new links to inflammation(18; 19). Also consistent with previous studies, adult CD dysbiosis was associated with species from the genus *Clostridium* including *C. neonatale* (SGB6169), *C. perfringens* (SGB6191), *C. butyricum* (SGB6170), and two lesser characterized species (AT4 (SGB4753) and 1001270J-160509-D11 (SGB6139))(14). Fewer taxa

were associated with UC (6) than CD (27), consistent with previous literature(14), but among UC associations, both *Blautia faecicola* (SGB4867) and *B. obeum* (SGB4811) were enriched.

23 positive associations—microbes present or enriched during disease—were unique to the pediatric cohort including associations between CD dysbiosis and the previously implicated species *Escherichia coli* (SGB10068), *Ruminococcus gnavus* (SGB4584), *Bacteroides fragilis* (SGB1855 and SGB1853), and *Clostridium symbiosum* (SGB4699)(20; 21; 14) (**Fig. 4B**). Interestingly, several species were enriched in pediatric CD dysbiosis that are typically found in probiotics including *Bifidobacterium breve* (SGB17247), *Bifidobacterium longum* (SGB17248), and *Lactobacillus acidophilus* (SGB7044)(22; 23; 24), along with a single SGB of *Faecalibacterium prausnitzii* (SGB15326, which was also associated with pediatric CD diagnosis).

By contrast with the small positive overlap, of the 223 negative associations that were significant in either adults or children, 53 (24%) of them overlapped including reductions in four *F. prausnitzii* species genome bins (SGB15316, SGB15318, SGB15332, SGB15342), *Phocaeicola vulgatus* (SGB1814), several *Bacteroides* species (*B. uniformis* (SGB1836), *B. ovatus* (SGB1871), *B. xylanisolvens* (SGB1867)), *Eubacterium rectale* (SGB4933), *Collinsella aerofaciens* (SGB14535), *Roseburia inulinivorans* (SGB4940), several *Alistipes* species (*A. shahii* (SGB2295), *A. putredinis* (SGB2318), *A. onderdonkii* (SGB2303)), *Blautia* species (*B. faecis* (SGB4820), *B. wexlerae* (SGB4837), and *B. massiliensis* (SGB4826)), and *Anaerostipes hadrus* (SGB4540). This confirms previous reports that typical gut commensals are lost during gastrointestinal inflammatory disorders(14; 25; 26; 27). Taken together, our findings confirm and expand our knowledge of IBD, differentiating both the gain and loss of specific microbes during inflammation, as well as the differences in these dysbioses among older and younger populations.

We sought to determine whether features of the gut ecosystem might link to the observation that the severity and progression of IBD tends to be worse in pediatric patients(28). As in most early onset chronic diseases, this is a setting in which causality can be difficult to determine: do gut microbiome changes drive this increased severity, or does more severe disease tend to manifest earlier in life? Perhaps the most relevant finding was that microbes typically hypothesized to drive inflammation were more likely to be enriched in the gut microbiome of adolescents. If gut microbial communities remain more plastic in adolescents than adults(10; 29), this adolescent-associated instability might allow invasion of opportunistic, fast-growing microbes more readily than in adults(30), thus forming a cause of more severe disease. This effect might be exacerbated by low fiber diets, a hallmark of children in the US(31), since recent studies have suggested that a shift from microbes utilizing carbohydrate to mucin was able to stimulate IBD phenotypes(32). This combination of early life gut instability, opportunistic invasion, and suboptimal diet could also have lasting impacts on the hosts' immune system(33). While the severity and faster progression of childhood IBD is likely multifaceted, these models suggest that gut instability and the invasion of IBD-typical microbes might play a more important role in pediatric IBD.

When MaAsLin 3 was run on the combined sample set from both age ranges, 372 significant associations were identified, of which 287 (77%) were prevalence associations. Of the 85 abundance associations, 50 were negative associations with either IBD diagnosis or gut dysbiosis, and, consistent with previous findings(34; 35), 89% (254) of the prevalence associations were negative. Since the sparsity was similar to the transcriptomic abundance data analyzed above (94.5% zeros in both), this suggests that in IBD, species themselves

might predominantly differ in prevalence (e.g. gain or loss), while pathway expressions (once controlled for functional potential) predominantly differ in abundance.

As expected, more taxa were lost in CD (121) than in UC (80), though many overlapped including *Bacteroides uniformis* (SGB1836), *Eubacterium rectale* (SGB4933), and many species in the *Roseburia* genus. In particular, the reduction in the prevalence of *Dysosmobacter welbionis* (SGB15078) in CD and UC dysbiosis (**Fig. 4C**) highlights the synergy between MetaPhlAn 4's taxonomic breadth and MaAsLin 3's model capabilities. *D. welbionis* is a recently isolated human gut commensal that is associated with metabolic disorders in humans and can prevent diet-induced obesity in mice(36; 37), but it is newly detectable in MetaPhlAn 4 relative to MetaPhlAn 3. When analyzed with MaAsLin 2, *D. welbionis* showed a negative abundance association with both UC and CD dysbiosis, but when analyzed with MaAsLin 3, this association was shown to be driven only by a reduced prevalence of *D. welbionis* during dysbiosis, not by a reduced abundance when present (prevalence $\beta_{\text{UC}} = -4.14$ (95% $\text{CI}_{\text{unadjusted}}: -5.86, -2.42), \beta_{\text{CD}} = -3.46$ ($-4.47, -2.46$); q-values $= 1.5 \times 10^{-4}, 5.1 \times 10^{-9}$; abundance $\beta_{\text{UC}} = -1.12$ $(-2.18, -0.06), \beta_{\text{CD}} =$ 0.15 $(-0.64, 0.95)$; q-values $= 0.24, 1$). Depending on the direction of causality, this may suggest that the organism's phenotypic effects are exerted when it is present regardless of abundance, or that it is so sensitive to an inflamed gut that it is driven below the limit of detection during IBD.

Relative and absolute coefficients differ by a constant shift when extraction efficiency is equal and all features are present

First, consider the case in which all features have equal extraction efficiency and there is no sparsity. Following the notation of ANCOM-BC, let $i \in \{1, ..., m\}$ be the feature index, $j \in \{1, ..., g\}$ be the covariate index, and $k \in \{1, ..., n\}$ be the sample index. Let $\mathbf{X}$ be the $n \times (g + 1)$ design matrix of the metadata (including the intercept). With A_{ik} as the absolute abundance of feature i in sample k, let Z_{ik} be the $\log_2$ absolute abundance, so $Z_{ik} = \log_2(A_{ik})$. Also, suppose the absolute abundance is related to the metadata by: $E(Z_{ik}) = \sum_j X_{kj}\beta_{ij} + \epsilon_{ik}$ where β_{ij} is the slope relating covariate j to feature i's $\log_2$ absolute abundance and ϵ_{ik} is some error with mean 0. These slopes can be grouped together for a feature as a $(g + 1) \times 1$ column vector: $\boldsymbol{\beta}_i = (\beta_{i0}, \beta_{i2}, ..., \beta_{ig})^T$. Let the $n \times 1$ vector $\mathbf{Z}_i = (Z_{i1}, Z_{i2}, ..., Z_{in})^T$ denote the vector of $\log_2$ absolute abundances for feature i. With $T_k = \sum_i A_{ik}$ as the total absolute abundance in sample k, let $D_k = \log_2(T_k)$. With $P_{ik} = A_{ik}/T_k$ as the relative abundance of feature i in sample k, let $Y_{ik} = \log_2(P_{ik})$, so $2^{Y_{ik}} = 2^{Z_{ik}}/2^{D_k} \iff Y_{ik} = Z_{ik} - D_k$. As with the absolute abundances, let $\mathbf{Y}_i$ be the $n \times 1$ vector of $\log_2$ relative abundances for feature i, and let $\mathbf{D}$ be the $n \times 1$ vector of $\log_2$ total abundances. Thus, $\mathbf{Y}_i = \mathbf{Z}_i - \mathbf{D}$ for all i.

When observing a vector $\mathbf{Z}_i$ and wanting to estimate $\boldsymbol{\beta}_i$, the ordinary least squares method is typically used: $\hat{\boldsymbol{\beta}}_i = (\mathbf{X}^T\mathbf{X})^{-1}\mathbf{X}^T\mathbf{Z}_i$. By the abundance decomposition above, this also gives:

$$\hat{\boldsymbol{\beta}}_i = (\mathbf{X}^T\mathbf{X})^{-1}\mathbf{X}^T\mathbf{Z}_i = (\mathbf{X}^T\mathbf{X})^{-1}\mathbf{X}^T\mathbf{Y}_i + (\mathbf{X}^T\mathbf{X})^{-1}\mathbf{X}^T\mathbf{D} = \hat{\boldsymbol{\beta}}_i^{\text{rel}} + \hat{\boldsymbol{\beta}}^{\text{tot}}$$

where $\hat{\boldsymbol{\beta}}_i^{\text{rel}}$ is the result of regressing the $\log_2$ relative abundances on the design matrix, and $\hat{\boldsymbol{\beta}}^{\text{tot}}$ is the result of regressing the $\log_2$ total abundances on the design matrix. Note that since $\hat{\boldsymbol{\beta}}^{\text{tot}}$ is the same for all features, if the absolute abundance coefficient for one feature i is d larger than the absolute abundance coefficient for another feature i' (i.e., $\hat{\beta}_{ij} - \hat{\beta}_{i'j} = d$), the relative abundance coefficient for feature i will be d larger than the

relative abundance coefficient for feature i' (i.e., $\hat{\beta}_{ij}^{\text{rel}} - \hat{\beta}_{i'j}^{\text{rel}} = d$). Thus, if all that is available is the relative abundance data, absolute slopes $\hat{\beta}_{ij}$ themselves cannot be determined. However, the relative coefficients $\hat{\beta}_{ij}^{\text{rel}}$ *can* be determined, and the ordering of and spacing between these coefficients will be identical to the ordering of and spacing between the absolute coefficient for each metadatum.

Since the OLS solution is unbiased for $\boldsymbol{\beta}_i$, the expectations will be equal too:

$$\boldsymbol{\beta}_i = E(\hat{\boldsymbol{\beta}}_i) = E(\hat{\boldsymbol{\beta}}_i^{\text{rel}}) + E(\hat{\boldsymbol{\beta}}^{\text{tot}}) = \boldsymbol{\beta}_i^{\text{rel}} + \boldsymbol{\beta}^{\text{tot}}.$$

Assuming at least half of the features do not change with respect to a particular metadatum (i.e., $\beta_{ij} = 0$ for at least half the features i), the median absolute abundance coefficient will be 0 (i.e., $\text{med}(\beta_{1j}, \beta_{2j}, ..., \beta_{mj}) = 0$), as has been previously noted in LOCOM(38). Expanding each of these coefficients using the expectation decomposition above gives

$$\text{med}(\beta_{1j}^{\text{rel}} + \beta_j^{\text{tot}}, \beta_{2j}^{\text{rel}} + \beta_j^{\text{tot}}, ..., \beta_{mj}^{\text{rel}} + \beta_j^{\text{tot}}) = 0.$$

Since the term β_j^{tot} is the same in all of these, this implies $\text{med}(\beta_{1j}^{\text{rel}}, \beta_{2j}^{\text{rel}}, ..., \beta_{mj}^{\text{rel}}) = -\beta_j^{\text{tot}}$. Thus, a test of $\beta_{ij} = 0$ is algebraically equivalent to the tests:

$$\beta_{ij}^{\text{rel}} + \beta_j^{\text{tot}} = 0 \iff \beta_{ij}^{\text{rel}} = \text{med}(\beta_{1j}^{\text{rel}}, \beta_{2j}^{\text{rel}}, ..., \beta_{mj}^{\text{rel}}).$$

That is, testing whether one feature's relative abundance slope for a metadatum is different from the median relative abundance slope for that metadatum is the same as testing whether that feature's absolute abundance slope is different from 0. This motivates the median comparison test implemented in MaAsLin 3.

Extraction efficiency

Now, let the extraction efficiency differ by feature, but assume each feature's extraction efficiency depends only on the feature, not on what else is in the sample. Let A_{ik} and Z_{ik} be the true absolute and log absolute abundances as above, and let E_i be the sampling efficiency of feature i with $S_i = \log_2(E_i)$. Now, let $T_k = \sum_i A_{ik}E_i$ and $D_k = \log_2(T_k)$, so $2^{D_k} = \sum_i 2^{Z_{ik}+S_i}$. Then, the relative abundances can be written as

$$P_{ik} = A_{ik}E_i/T_k \iff 2^{Y_{ik}} = 2^{Z_{ik}}2^{S_i}/2^{D_k} \iff Y_{ik} = Z_{ik} + S_i - D_k \iff Z_{ik} = Y_{ik} + D_k - S_i.$$

Let $\mathbf{S}_i$ be S_i repeated p times to match the dimensions of the feature-specific slopes. With an equivalent decomposition to before,

$$\hat{\boldsymbol{\beta}}_i = (\mathbf{X}^T\mathbf{X})^{-1}\mathbf{X}^T\mathbf{Z}_i = (\mathbf{X}^T\mathbf{X})^{-1}\mathbf{X}^T\mathbf{Y}_i + (\mathbf{X}^T\mathbf{X})^{-1}\mathbf{X}^T\mathbf{D} - (\mathbf{X}^T\mathbf{X})^{-1}\mathbf{X}^T\mathbf{S}_i = \hat{\boldsymbol{\beta}}_i^{\text{rel}} + \hat{\boldsymbol{\beta}}^{\text{tot}} - \hat{\boldsymbol{\beta}}_i^{\text{eff}}$$

where $\hat{\boldsymbol{\beta}}_i^{\text{eff}}$ is the regression of the $\log_2$ sampling efficiency on the covariates $\mathbf{X}$. However, since all the elements of $\mathbf{S}_i$ are equal, $\hat{\boldsymbol{\beta}}_i^{\text{eff}}$ will just be an intercept and then zeros for all the slopes corresponding to covariates (i.e., $\hat{\boldsymbol{\beta}}_i^{\text{eff}} = [\hat{\beta}_{i0}, 0, ..., 0]^T$). Thus, excluding the intercept, the same result as before holds: $\hat{\boldsymbol{\beta}}_i = \hat{\boldsymbol{\beta}}_i^{\text{rel}} + \hat{\boldsymbol{\beta}}^{\text{tot}}$. Therefore, the same results as above follow, justifying the median comparison technique and its equivalence to absolute testing when at least half the features are unassociated with a metadatum.

With sparsity

For simplicity, consider the equal extraction efficiency case again. Before, the coefficients could be decomposed as $\hat{\boldsymbol{\beta}}_i = \hat{\boldsymbol{\beta}}_i^{\text{rel}} + \hat{\boldsymbol{\beta}}^{\text{tot}}$ where $\hat{\boldsymbol{\beta}}^{\text{tot}}$ was the same for all features, but this was reliant on the data matrix $\mathbf{X}$ being the same for all features. When some features have zero abundances and only non-zero abundances are included in the regression, there will be different matrices $\mathbf{X}^{(i)}$ for each feature. Thus, the equation relating the slopes is $\hat{\boldsymbol{\beta}}_i = \hat{\boldsymbol{\beta}}_i^{\text{rel}} + \hat{\boldsymbol{\beta}}_i^{\text{tot}}$ with an index on the total abundance regression coefficient. Since $\hat{\boldsymbol{\beta}}_i^{\text{tot}}$ can now differ by feature, the ordering and spacing of the $\hat{\boldsymbol{\beta}}_i$ are not necessarily the same as of the $\hat{\boldsymbol{\beta}}_i^{\text{rel}}$. To proceed, note that the following quantities are being estimated with these regressions:

$$\begin{aligned}
\boldsymbol{\beta}_i = E(\hat{\boldsymbol{\beta}}_i) &= E((\mathbf{X}^T\mathbf{X})^{-1}\mathbf{X}^T\mathbf{Z}_i|\mathbf{X}, A_{ik} > 0\ \forall k) \\
\boldsymbol{\beta}_i^{\text{rel}} = E(\hat{\boldsymbol{\beta}}_i^{\text{rel}}) &= E((\mathbf{X}^T\mathbf{X})^{-1}\mathbf{X}^T\mathbf{Y}_i|\mathbf{X}, A_{ik} > 0\ \forall k) \\
\boldsymbol{\beta}_i^{\text{tot}} = E(\hat{\boldsymbol{\beta}}_i^{\text{tot}}) &= E((\mathbf{X}^T\mathbf{X})^{-1}\mathbf{X}^T\mathbf{D}|\mathbf{X}, A_{ik} > 0\ \forall k)
\end{aligned}$$

Since $\boldsymbol{\beta}_i = \boldsymbol{\beta}_i^{\text{rel}} + \boldsymbol{\beta}_i^{\text{tot}}$, we would retain the consistent ordering and spacing properties, at least in expectation, if

$$E((\mathbf{X}^T\mathbf{X})^{-1}\mathbf{X}^T\mathbf{D}|\mathbf{X}, A_{ik} > 0\ \forall k) = E((\mathbf{X}^T\mathbf{X})^{-1}\mathbf{X}^T\mathbf{D}|\mathbf{X}) \iff E(\mathbf{D}|\mathbf{X}, A_{ik} > 0\ \forall k) = E(\mathbf{D}|\mathbf{X})$$

for all i. That is, the results before would hold if (1) all features are always present (no sparsity) or (2) the expected total abundance is independent of whether any particular feature is actually present (i.e., the conditional expectation of $\mathbf{D}$ is the same with or without the conditioning on $A_{ik} > 0\ \forall k$). When the carrying capacity of a community is determined by the covariates and the community is at carrying capacity (e.g., in an adult gut), this is more likely to be true. However, during periods of colonization (e.g., in the infant gut), this is unlikely to hold.

Test implementation

The test statistic is $\frac{\hat{\beta}_{ij}^{\text{rel}} - \hat{M}_j^{\text{rel}}}{\sqrt{\text{Var}\left(\hat{\beta}_{ij}^{\text{rel}} - \hat{M}_j^{\text{rel}}\right)}}$. The variance can be decomposed: $\text{Var}\left(\hat{\beta}_{ij}^{\text{rel}} - \hat{M}_j^{\text{rel}}\right) = \text{Var}\left(\hat{\beta}_{ij}^{\text{rel}}\right) + \text{Var}\left(\hat{M}_j^{\text{rel}}\right) - 2\text{Cov}(\hat{\beta}_{ij}^{\text{rel}}, \hat{M}_j^{\text{rel}})$. First, $\text{Var}\left(\hat{\beta}_{ij}^{\text{rel}}\right)$ is already estimated in the model fitting. Second, $\text{Var}\left(\hat{M}_j^{\text{rel}}\right)$ is estimated as $\frac{1}{4n(f_{\hat{\beta}_{ij}}(0))^2} = \frac{2\pi\widehat{\text{Var}_i(\hat{\beta}_{ij})}}{4n}$ based on the asymptotic distribution of the median assuming the $\hat{\beta}_{ij}$ are i.i.d. from a normal distribution with mean 0 (39). While this will almost always be violated practically, the estimate of the $f_{\hat{\beta}_{ij}}(0)$ will typically be too small (the estimated density will be too dispersed), resulting in a conservative rather than anti-conservative test. Third, $\text{Cov}(\hat{\beta}_{ij}^{\text{rel}}, \hat{M}_j^{\text{rel}})$ is estimated by bootstrapping the $\hat{\beta}_{ij}^{\text{rel}}$ from their approximate normal distributions and computing $\hat{M}_j^{\text{rel}}$. The computed test statistic is then evaluated against the distribution of $\hat{\beta}_{ij}^{\text{rel}}$ to determine a p-value. While the distribution will not be exact, it will typically be close to a standard normal.

Supplementary Figures

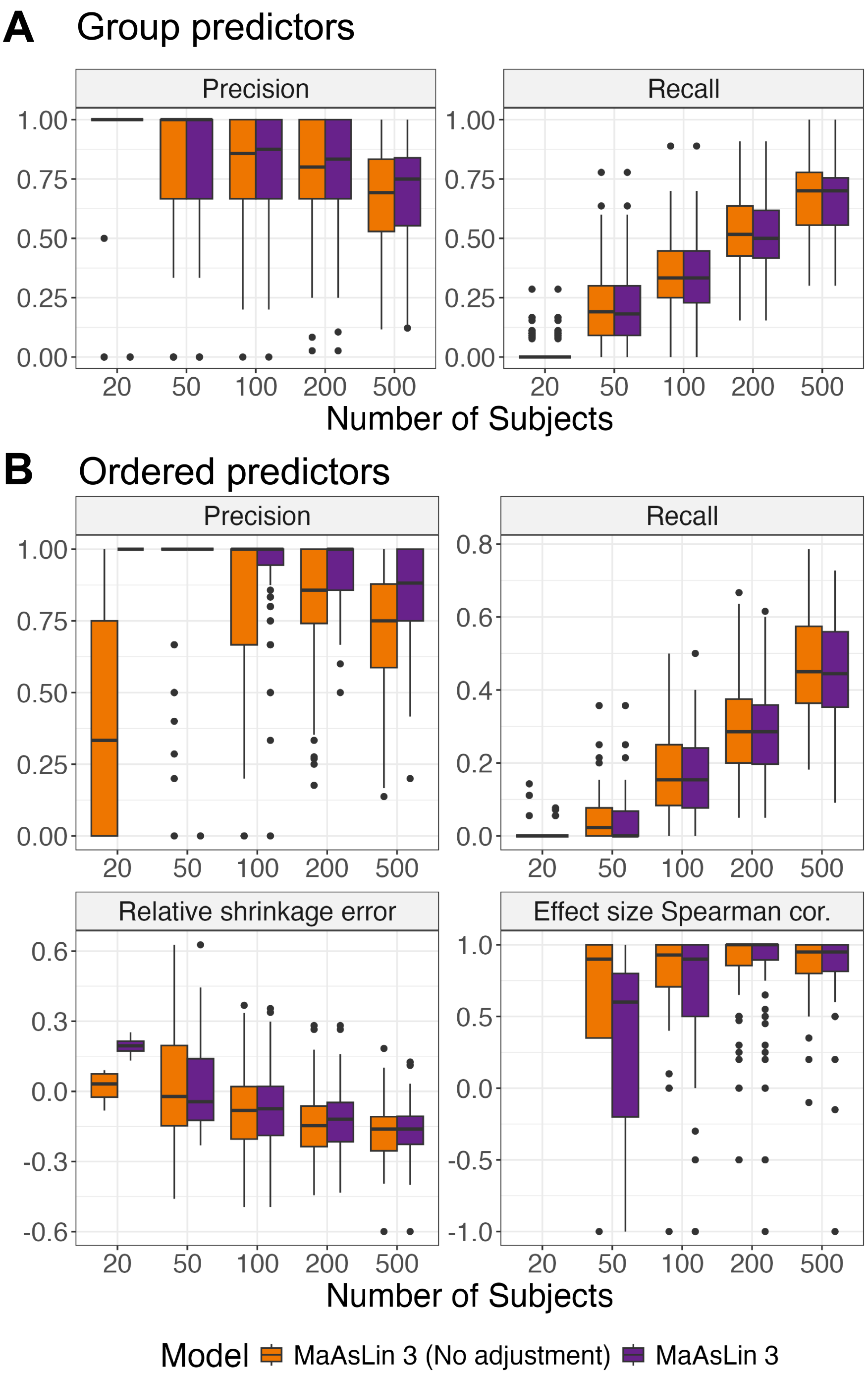

Supplementary Figure 1: MaAsLin 3 enables inference for group-wise differences and ordered predictors. MaAsLin 3 was run on 100 synthetic log-normal datasets from SparseDOSSA 2. For these simulations, 100 features and 2 metadata (one continuous or binary and one a categorical variable for group-wise or ordered predictors) were simulated with 10% of the feature-metadatum pairs having true associations. Coefficients for the continuous or binary variable were chosen from 2.5 to 5 uniformly. For the group-wise and ordered predictors, a value was chosen from 2.5 to 5 uniformly to represent the most extreme group's (level's) difference from baseline, and this value was subdivided according to a Dirichlet(1) distribution to obtain the coefficients for the other groups (levels). Half of the associations were positive; the rest were negative. Half were abundance associations; the rest were prevalence associations. The read depth (analogous to 16S read count) per sample was drawn from a log-normal distribution with a mean of 50,000. Significant associations (q-value less than 0.1, joint q-value for MaAsLin 3) were considered correct if they matched the true associations in the feature, metadatum, and type of association (prevalence/abundance). The metrics were calculated as before. 1 is optimal for all metrics except shrinkage, for which 0 is optimal. Each point represents a simulated dataset. Only the group predictor (**A**) or ordered predictor (**B**) variables were evaluated for accuracy. MaAsLin 3 was run with and without the median compositionality adjustment in both cases. Each point represents a simulated dataset. Boxplots display the median, interquartile range, and whiskers extending to the most extreme values within 1.5×IQR and individual points indicating outliers.

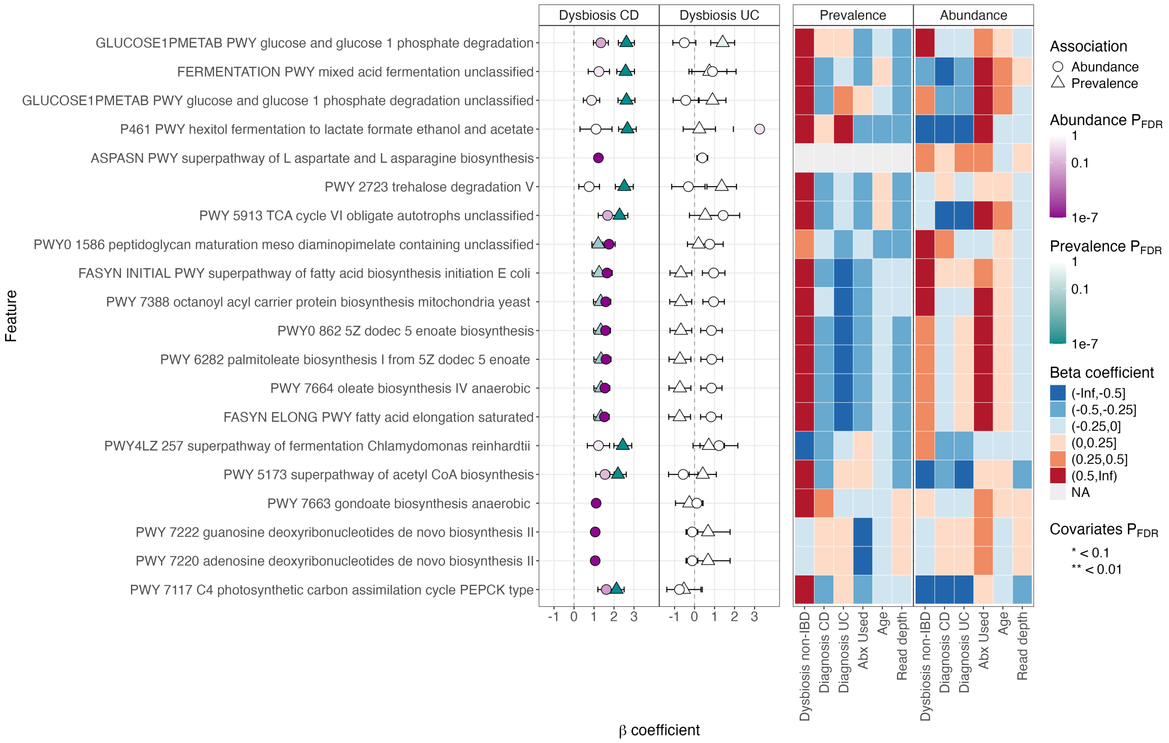

Supplementary Figure 2: MaAsLin 3 applied to the HMP2 metatranscriptomics data verifies and extends previous findings. The metatranscriptomics pathway abundances from the HMP2 cohort were regressed in MaAsLin 3 using a model that incorporated disease-stratified dysbiosis, disease diagnosis, antibiotic usage, age, read depth, a per-participant random intercept, and the pathway's metagenomic abundance as a feature-specific covariate.

Error bars represent the coefficients' standard errors.

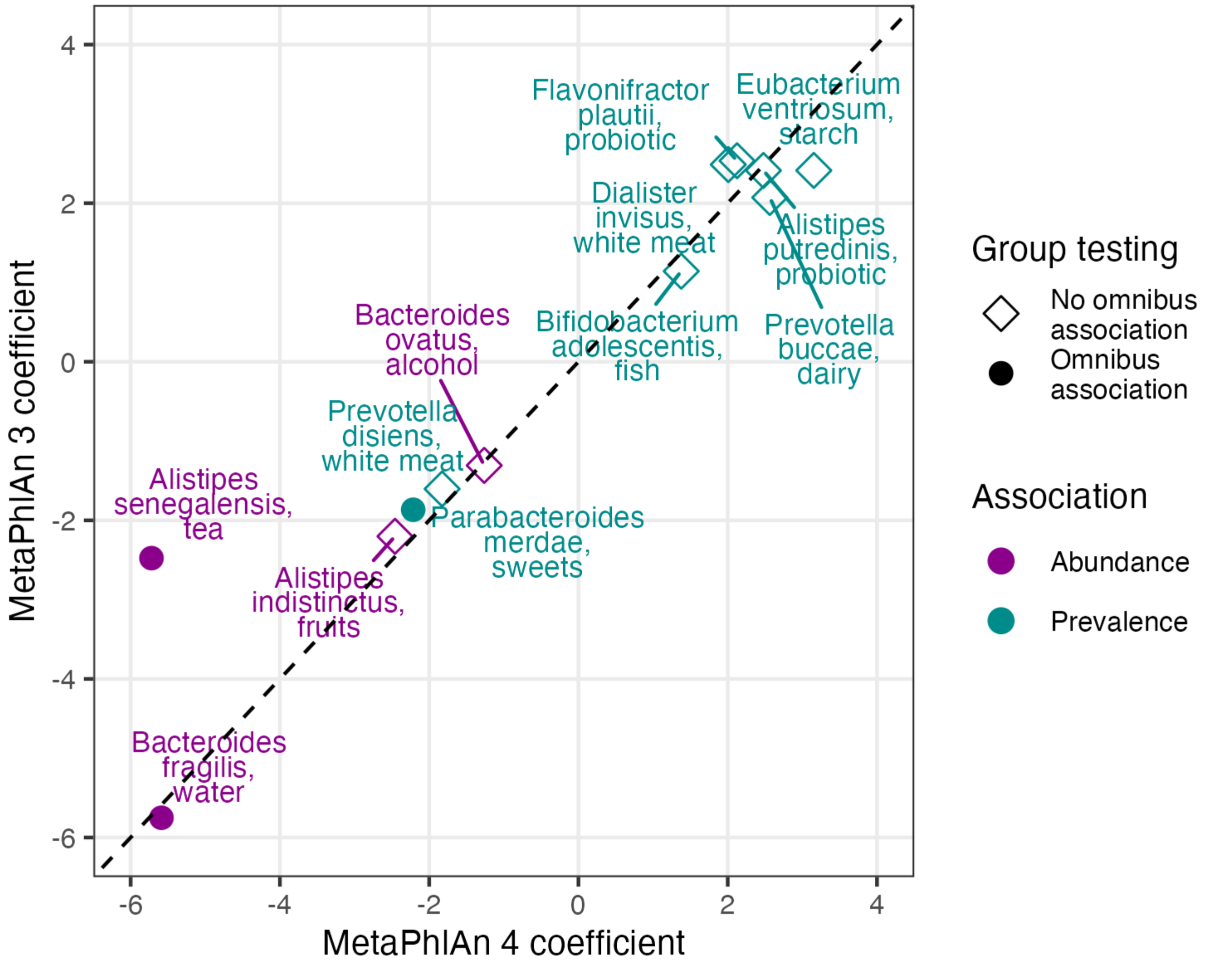

Supplementary Figure 3. Significant associations in both MetaPhlAn 3 and MetaPhlAn 4 profiles largely agree in fit coefficients. Using the subset of HMP2 participants with CD, abundances were regressed in MaAsLin 3 using a model that incorporated categorical dietary frequency information as a group or ordered predictor along with dysbiosis, antibiotic usage, age, read depth, and a per-participant random intercept. Associations identified as significant using both the MetaPhlAn 3 and MetaPhlAn 4 profiles are plotted.

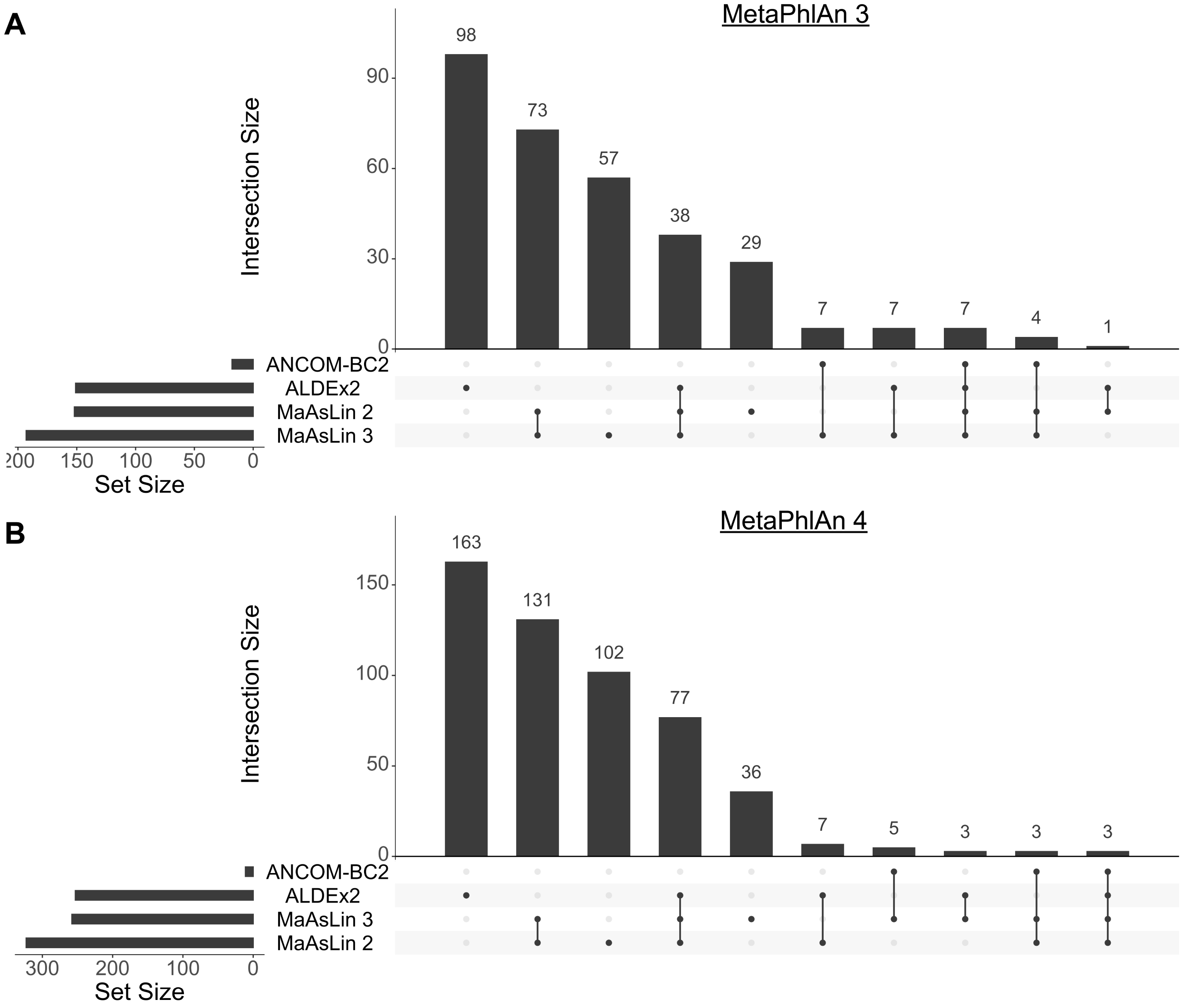

Supplementary Figure 4: Most IBD associations discovered by MaAsLin 3 overlapped with other methods. The species-level abundances from the HMP2 cohort profiled with MetaPhlAn 3 (**A**) or MetaPhlAn 4 (**B**) were regressed in each method using a model that incorporated disease-stratified dysbiosis, disease diagnosis, antibiotic usage, age, read depth, and a per-participant random intercept (or, for ALDEx2, a fixed intercept subsequently removed from analysis). Because of the possibility for false positives identified in the simulations, only significant (q-value ¡0.1) coefficients with absolute values greater than 1 were evaluated for their overlap.

References

[1] Kumar, M. S. *et al.* Analysis and correction of compositional bias in sparse sequencing count data. *BMC genomics* **19**, 799 (2018).

[2] Zhu, Z., Satten, G. A. & Hu, Y.-J. Integrative analysis of relative abundance data and presence-absence data of the microbiome using the LDM. *Bioinformatics (Oxford, England)* **38**, 2915–2917 (2022).

[3] Zhang, Y., Thompson, K. N., Huttenhower, C. & Franzosa, E. A. Statistical approaches for differential expression analysis in metatranscriptomics. *Bioinformatics (Oxford, England)* **37**, i34–i41 (2021).

[4] Lloyd-Price, J. *et al.* Multi-omics of the gut microbial ecosystem in inflammatory bowel diseases. *Nature* **569**, 655–662 (2019).

[5] Beghini, F. *et al.* Integrating taxonomic, functional, and strain-level profiling of diverse microbial communities with bioBakery 3. *eLife* **10**, e65088 (2021).

[6] Blanco-Míguez, A. *et al.* Extending and improving metagenomic taxonomic profiling with uncharacterized species using MetaPhlAn 4. *Nature Biotechnology* **41**, 1633–1644 (2023).

[7] Duncan, S. H. *et al.* Reduced dietary intake of carbohydrates by obese subjects results in decreased concentrations of butyrate and butyrate-producing bacteria in feces. *Applied and Environmental Microbiology* **73**, 1073–1078 (2007).

[8] Russell, W. R. *et al.* High-protein, reduced-carbohydrate weight-loss diets promote metabolite profiles likely to be detrimental to colonic health. *The American Journal of Clinical Nutrition* **93**, 1062–1072 (2011).

[9] Ghosh, T. S., Das, M., Jeffery, I. B. & O'Toole, P. W. Adjusting for age improves identification of gut microbiome alterations in multiple diseases. *eLife* **9**, e50240 (2020).

[10] Agans, R. *et al.* Distal gut microbiota of adolescent children is different from that of adults. *FEMS microbiology ecology* **77**, 404–412 (2011).

[11] Federici, S. *et al.* Targeted suppression of human IBD-associated gut microbiota commensals by phage consortia for treatment of intestinal inflammation. *Cell* **185**, 2879–2898.e24 (2022).

[12] Zhang, Q. *et al.* Klebsiella pneumoniae Induces Inflammatory Bowel Disease Through Caspase-11-Mediated IL18 in the Gut Epithelial Cells. *Cellular and Molecular Gastroenterology and Hepatology* **15**, 613–632 (2023).

[13] Higgins, L. M., Frankel, G., Douce, G., Dougan, G. & MacDonald, T. T. Citrobacter rodentium infection in mice elicits a mucosal Th1 cytokine response and lesions similar to those in murine inflammatory bowel disease. *Infection and Immunity* **67**, 3031–3039 (1999).

[14] Franzosa, E. A. *et al.* Gut microbiome structure and metabolic activity in inflammatory bowel disease. *Nature Microbiology* **4**, 293–305 (2019).

[15] Seishima, J. *et al.* Gut-derived Enterococcus faecium from ulcerative colitis patients promotes colitis in a genetically susceptible mouse host. *Genome Biology* **20**, 252 (2019).

[16] Zhou, Y. *et al.* Increased Enterococcus faecalis infection is associated with clinically active Crohn disease. *Medicine* **95**, e5019 (2016).

[17] Rashid, T., Ebringer, A. & Wilson, C. The role of Klebsiella in Crohn's disease with a potential for the use of antimicrobial measures. *International Journal of Rheumatology* **2013**, 610393 (2013).

[18] Santiago, A. *et al.* Crohn's disease proteolytic microbiota enhances inflammation through PAR2 pathway in gnotobiotic mice. *Gut Microbes* **15**, 2205425 (2023).

[19] Huang, Z., Wang, C., Huang, Q., Yan, Z. & Yin, Z. Hungatella hathewayi impairs the sensitivity of colorectal cancer cells to 5-FU through decreasing CDX2 expression. *Human Cell* **36**, 2055–2065 (2023).

[20] Gevers, D. *et al.* The treatment-naive microbiome in new-onset Crohn's disease. *Cell Host & Microbe* **15**, 382–392 (2014).

[21] Rabizadeh, S. *et al.* Enterotoxigenic bacteroides fragilis: a potential instigator of colitis. *Inflammatory Bowel Diseases* **13**, 1475–1483 (2007).

[22] Bozzi Cionci, N., Baffoni, L., Gaggìa, F. & Di Gioia, D. Therapeutic Microbiology: The Role of Bifidobacterium breve as Food Supplement for the Prevention/Treatment of Paediatric Diseases. *Nutrients* **10**, 1723 (2018).

[23] Yao, S., Zhao, Z., Wang, W. & Liu, X. Bifidobacterium Longum: Protection against Inflammatory Bowel Disease. *Journal of Immunology Research* **2021**, 8030297 (2021).

[24] Kailasapathy, K. & Chin, J. Survival and therapeutic potential of probiotic organisms with reference to Lactobacillus acidophilus and Bifidobacterium spp. *Immunology and Cell Biology* **78**, 80–88 (2000).

[25] Imhann, F. *et al.* Interplay of host genetics and gut microbiota underlying the onset and clinical presentation of inflammatory bowel disease. *Gut* **67**, 108–119 (2018).

[26] Huttenhower, C., Kostic, A. D. & Xavier, R. J. Inflammatory bowel disease as a model for translating the microbiome. *Immunity* **40**, 843–854 (2014).

[27] Sokol, H. & Seksik, P. The intestinal microbiota in inflammatory bowel diseases: time to connect with the host. *Current Opinion in Gastroenterology* **26**, 327–331 (2010).

[28] Van Limbergen, J. *et al.* Definition of phenotypic characteristics of childhood-onset inflammatory bowel disease. *Gastroenterology* **135**, 1114–1122 (2008).

[29] Hollister, E. B. *et al.* Structure and function of the healthy pre-adolescent pediatric gut microbiome. *Microbiome* **3**, 36 (2015).

[30] Kumbhari, A. *et al.* Discovery of disease-adapted bacterial lineages in inflammatory bowel diseases. *Cell Host & Microbe* **32**, 1147–1162.e12 (2024).

[31] Ahluwalia, N. *et al.* Usual nutrient intakes of US infants and toddlers generally meet or exceed Dietary Reference Intakes: findings from NHANES 2009-2012. *The American Journal of Clinical Nutrition* **104**, 1167–1174 (2016).

[32] Pereira, G. V. *et al.* Opposing diet, microbiome, and metabolite mechanisms regulate inflammatory bowel disease in a genetically susceptible host. *Cell Host & Microbe* **32**, 527–542.e9 (2024).

[33] Metcalf, C. J. E. & Koskella, B. Protective microbiomes can limit the evolution of host pathogen defense. *Evolution Letters* **3**, 534–543 (2019).

[34] Vester-Andersen, M. K. *et al.* Increased abundance of proteobacteria in aggressive Crohn's disease seven years after diagnosis. *Scientific Reports* **9**, 13473 (2019).

[35] Moustafa, A. *et al.* Genetic risk, dysbiosis, and treatment stratification using host genome and gut microbiome in inflammatory bowel disease. *Clinical and Translational Gastroenterology* **9**, e132 (2018).

[36] Le Roy, T. *et al.* Dysosmobacter welbionis is a newly isolated human commensal bacterium preventing diet-induced obesity and metabolic disorders in mice. *Gut* **71**, 534–543 (2022).

[37] Moens de Hase, E. *et al.* Impact of metformin and Dysosmobacter welbionis on diet-induced obesity and diabetes: from clinical observation to preclinical intervention. *Diabetologia* **67**, 333–345 (2024).

[38] Hu, Y., Satten, G. A. & Hu, Y.-J. LOCOM: A logistic regression model for testing differential abundance in compositional microbiome data with false discovery rate control. *Proceedings of the National Academy of Sciences of the United States of America* **119**, e2122788119 (2022).

[39] Casella, G. & Berger, R. *Statistical Inference* (CRC Press, 2024). Google-Books-ID: cqUIEQAAQBAJ.
