# Supplementary material for: MaAsLin 3: refining and extending generalized multivariable linear models for meta-omic association discovery
Source: Nat Methods. 2026 Jan 15;23(3):554–64. doi: 10.1038/s41592-025-02923-9 (PMC12982127; doi:10.1038/s41592-025-02923-9)
Supplement: Supplementary file 2 — Reporting Summary [file 41592_2025_2923_MOESM2_ESM.pdf]

nature portfolio

Corresponding author(s): Curtis Huttenhower

Last updated by author(s): May 28, 2025

Reporting Summary

Nature Portfolio wishes to improve the reproducibility of the work that we publish. This form provides structure for consistency and transparency in reporting. For further information on Nature Portfolio policies, see our Editorial Policies and the Editorial Policy Checklist.

Statistics

For all statistical analyses, confirm that the following items are present in the figure legend, table legend, main text, or Methods section.

n/a	Confirmed	
☐	☒	The exact sample size (*n*) for each experimental group/condition, given as a discrete number and unit of measurement
☐	☒	A statement on whether measurements were taken from distinct samples or whether the same sample was measured repeatedly
☐	☒	The statistical test(s) used AND whether they are one- or two-sided<br>*Only common tests should be described solely by name; describe more complex techniques in the Methods section.*
☐	☒	A description of all covariates tested
☐	☒	A description of any assumptions or corrections, such as tests of normality and adjustment for multiple comparisons
☐	☒	A full description of the statistical parameters including central tendency (e.g. means) or other basic estimates (e.g. regression coefficient) AND variation (e.g. standard deviation) or associated estimates of uncertainty (e.g. confidence intervals)
☐	☒	For null hypothesis testing, the test statistic (e.g. *F*, *t*, *r*) with confidence intervals, effect sizes, degrees of freedom and *P* value noted<br>*Give P values as exact values whenever suitable.*
☒	☐	For Bayesian analysis, information on the choice of priors and Markov chain Monte Carlo settings
☒	☐	For hierarchical and complex designs, identification of the appropriate level for tests and full reporting of outcomes
☐	☒	Estimates of effect sizes (e.g. Cohen's *d*, Pearson's *r*), indicating how they were calculated

Our web collection on statistics for biologists contains articles on many of the points above.

Software and code

Policy information about availability of computer code

Data collection	Datasets were manually downloaded from their source locations (see Data) with no code.
Data analysis	All code used to analyze data is provided at https://github.com/WillNickols/maaslin3_benchmark/. The versions of software used in the analysis are: R (4.3.0), MaAsLin 3 (3.0.12), ALDEx2 (1.36.0), ANCOM-BC2 (2.4.0), MaAsLin 2 (1.16.0), DESeq2 (1.44.0), edgeR (4.2.2), SparseDOSSA (0.99.2).

For manuscripts utilizing custom algorithms or software that are central to the research but not yet described in published literature, software must be made available to editors and reviewers. We strongly encourage code deposition in a community repository (e.g. GitHub). See the Nature Portfolio guidelines for submitting code & software for further information.

Data

Policy information about availability of data

All manuscripts must include a data availability statement. This statement should provide the following information, where applicable:

- Accession codes, unique identifiers, or web links for publicly available datasets
- A description of any restrictions on data availability
- For clinical datasets or third party data, please ensure that the statement adheres to our policy

The 38 real datasets used in the randomization procedure are available at https://figshare.com/articles/dataset/16S_rRNA_Microbiome_Datasets/14531724. The metadata and bioBakery 3 outputs for the IBDMDB data are available at https://ibdmdb.org/downloads/html/products_MGX_2017-08-12.html (Merged Table tab,

taxonomic_profiles_3.tsv.gz and pathabundances_3.tsv.gz files). All other data including the synthetic datasets, the IBDMDB MetaPhlAn 4 profiles, and the three datasets with inferred absolute abundance data are available at https://figshare.com/s/8c09e0f276b427f07a.

Research involving human participants, their data, or biological material

Policy information about studies with human participants or human data. See also policy information about sex, gender (identity/presentation), and sexual orientation and race, ethnicity and racism.

Reporting on sex and gender	N/A
Reporting on race, ethnicity, or other socially relevant groupings	N/A
Population characteristics	N/A
Recruitment	N/A
Ethics oversight	N/A

Note that full information on the approval of the study protocol must also be provided in the manuscript.

Field-specific reporting

Please select the one below that is the best fit for your research. If you are not sure, read the appropriate sections before making your selection.

☒ Life sciences ☐ Behavioural & social sciences ☐ Ecological, evolutionary & environmental sciences

For a reference copy of the document with all sections, see nature.com/documents/nr-reporting-summary-flat.pdf

Life sciences study design

All studies must disclose on these points even when the disclosure is negative.

Sample size

Infant gut dataset: 178 infants, 650 samples
Mouse diet dataset: 12 mice, 45 samples
IBD/PSC dataset: 170 participants, 170 samples
IBDMDB: 130 participants, 1637 samples

The inclusion of 4 datasets with 2502 samples was deemed sufficient because the included datasets capture a diverse range of topics relevant to human health, and the number and size of included datasets is similar to or greater than that of other similar methods development and evaluation publications.

Data exclusions

In the IBD/PSC dataset, samples with missingness in diagnosis, age, gender, BMI, FC, or CRP were excluded to avoid introducing complexities when comparing across differential abundance tools while simultaneously dealing with missing data and because proper clinical effects were not the intention of this section. Likewise, participants with a UC diagnosis only (4 after earlier filtering) were excluded because there were too few of these participants to make accurate comparisons between differential abundance tools.

Replication

Reproducible workflows for the evaluations are available at https://github.com/WillNickols/maaslin3_benchmark/. The analysis of 38 binary datasets was performed as in Nearing et al. 2022, and the findings were similar (independent replication of previous results). The IBDMDB analyses were performed with both MaAsLin 3 and MaAsLin 2 since MaAsLin 2 had been applied to these data beforehand, and the results were largely consistent (independent replication of previous results). For all other analyses, simulations were performed 100 times each and seeds were set for exact reproducibility, but no independent replication was performed.

Randomization

No randomization was performed since no causal claims were addressed. For the infant dataset, the linear model included days since birth and read depth as fixed effects and infant ID as a random effect. For the mouse diet dataset, the model included diet and day as fixed effects and mouse ID as a random effect (read depth was not included because all samples had equal read depths). For the IBD/PSC dataset, diagnosis, age, gender, BMI, and read depth were included as fixed effects with diagnosis as a categorical variable that compared PSC-only, PSC-UC, CD-only, and PSC-CD against healthy controls as the baseline. Read depth was included as a covariate in the real datasets because deeper sequencing will often associate with higher prevalence (taxa are detected more often with more reads), and this deeper sequencing could be confounded with the key sample covariates.

The IBDMDB dataset was analyzed with a linear model incorporating antibiotic usage, diagnosis, and dysbiosis status as fixed effects and participant ID as a random effect. When not already split by pediatric and adult populations, age was also included as a fixed effect. Using the subset of samples from participants with CD, abundances were regressed using a model that incorporated categorical dietary frequency information as a group or ordered predictor. Also included in this model were dysbiosis, antibiotic usage, age, and read depth as fixed effects and participant ID as a random intercept. Metatranscriptomic relative abundances were regressed on age, antibiotic usage, diagnosis, and dysbiosis status as fixed effects, participant ID as a random effect, and pathway DNA relative abundance as a covariate-specific fixed effect.

Blinding

Blinding was not relevant since all datasets were previously published.

Reporting for specific materials, systems and methods

We require information from authors about some types of materials, experimental systems and methods used in many studies. Here, indicate whether each material, system or method listed is relevant to your study. If you are not sure if a list item applies to your research, read the appropriate section before selecting a response.

Materials & experimental systems

n/a	Involved in the study
☒	☐ Antibodies
☒	☐ Eukaryotic cell lines
☒	☐ Palaeontology and archaeology
☒	☐ Animals and other organisms
☒	☐ Clinical data
☒	☐ Dual use research of concern
☒	☐ Plants

Methods

n/a	Involved in the study
☒	☐ ChIP-seq
☒	☐ Flow cytometry
☒	☐ MRI-based neuroimaging

Plants

Seed stocks

Report on the source of all seed stocks or other plant material used. If applicable, state the seed stock centre and catalogue number. If plant specimens were collected from the field, describe the collection location, date and sampling procedures.

Novel plant genotypes

Describe the methods by which all novel plant genotypes were produced. This includes those generated by transgenic approaches, gene editing, chemical/radiation-based mutagenesis and hybridization. For transgenic lines, describe the transformation method, the number of independent lines analyzed and the generation upon which experiments were performed. For gene-edited lines, describe the editor used, the endogenous sequence targeted for editing, the targeting guide RNA sequence (if applicable) and how the editor was applied.

Authentication

Describe any authentication procedures for each seed stock used or novel genotype generated. Describe any experiments used to assess the effect of a mutation and, where applicable, how potential secondary effects (e.g. second site T-DNA insertions, mosiacism, off-target gene editing) were examined.
